# Risk Factors for Development and Severity of COVID-19 in COPD Patients

**DOI:** 10.3389/fmed.2021.714570

**Published:** 2021-08-09

**Authors:** Matteo Bonato, Umberto Semenzato, Mariaenrica Tinè, Erica Bazzan, Marco Damin, Davide Biondini, Alvise Casara, Micaela Romagnoli, Graziella Turato, Manuel G. Cosio, Marina Saetta, Simonetta Baraldo

**Affiliations:** ^1^Department of Cardiac, Thoracic, Vascular Sciences, and Public Health, University of Padova, Padova, Italy; ^2^Pulmonology Unit, Ospedale Ca' Foncello, Azienda Unità Locale Socio Sanitaria 2 (AULSS2) Marca Trevigiana, Treviso, Italy; ^3^Respiratory Division, Meakins-Christie Laboratories, McGill University, Montreal, QC, Canada

**Keywords:** COVID-19, COPD comorbidities, clinical outcome, emphysema, DLCO

## Abstract

The impact that COVID-19 could have on patients with COPD is a real concern. In this study we evaluated, in a cohort of longitudinally followed COPD subjects, the incidence of COVID-19, seeking for possible risk factors and prognostic factors predicting the clinical outcome. In our cohort of 370 patients (followed for 5.3 ± 2.7 years), 22 developed COVID-19 (COPD/COVID-19+) between February/November 2020 (5.9%). Cardio-metabolic conditions (hypertension, dyslipidemia, obesity, diabetes) but not respiratory abnormalities (FEV_1_, DLCO, emphysema and exacerbation history), were risk factors for development of COVID-19 in COPD patients. Out of the 22 COPD/COVID-19+ patients, 10 needed intensive care. Low DLCO and emphysema, but also metabolic comorbidities, were related to the need for intensive care.

## Introduction

Coronavirus disease 2019 (COVID-19), caused by the SARS-CoV-2, emerged in late 2019 in China and subsequently became a pandemic. SARS-CoV-2 may lead to pneumonia and respiratory failure. In Chronic Obstructive Pulmonary Disease (COPD) the underlying lung abnormalities, along with impairment of the immune responses to respiratory infections (1), might make these patients more susceptible to the development and severity of COVID-19.

The incidence of COPD in COVID-19 cohorts has been reported to range from 0 to 10% in China and 5.6–9.2% in Italy (2), although how the diagnosis of COPD was made was often unclear (3). However, the risk of patients with known COPD for the development of COVID-19 and its morbid consequences have not been clearly investigated. Underlying COPD may lead to respiratory failure, need for Intensive Care Unit (ICU) admission, mechanical ventilation or death (2).

This study was designed to: 1) evaluate the incidence of COVID-19 in a cohort of known COPD subjects followed longitudinally; 2) study the possible risk factors for the development of COVID-19; 3) identify the pathophysiological factors influencing the clinical outcome.

## Methods

The cohort included 370 subjects with COPD, diagnosed according to GOLD 2020 (4), all routinely followed in the COPD clinics at Padova University Hospital and Treviso City Hospital, Italy. All subjects provided written consent. The mean follow-up duration was 5.3 ± 2.7 years (date of first inclusion October 11th, 2012; date of last inclusion February 12th, 2019). Subjects with asthma or history of asthma were excluded. Subjects underwent yearly clinical evaluation including pulmonary function tests and blood cell count. High-resolution computed tomography (HRCT) was done at diagnosis. Annual frequency of exacerbations was recorded (4). We considered as COVID-19 cases all patients of the cohort who presented at the two public hospitals for symptomatic COVID-19 from February 21st until November 14th, 2020, whose SARS-CoV-2 infection was confirmed by real-time reverse transcription polymerase chain reaction from a nasopharyngeal swab/tracheal aspirate. Notification of cases was obtained by consultation of the electronic registry of all patients of the COPD cohort; inquiries were repeated every 4 weeks until November 14th, 2020. Clinical and functional data refer to the last follow-up visit for each patient before the pandemic outbreak.

In patients who developed symptomatic COVID-19, the following clinical outcomes were considered: disease severity, defined as the need for intensive care (ICU), length of hospitalization, in-hospital mortality and mortality at 6-months. Mann-Whitney, χ^2^ and Fisher's exact-test were performed for comparisons between groups, as appropriate; bivariate correlations were estimated using Spearman's rank-correlation coefficient. Statistics were performed using SPSS (v26, IBM Armonk, NY, USA) (level of significance *p* < 0.05).

## Results

**Figure 1:**
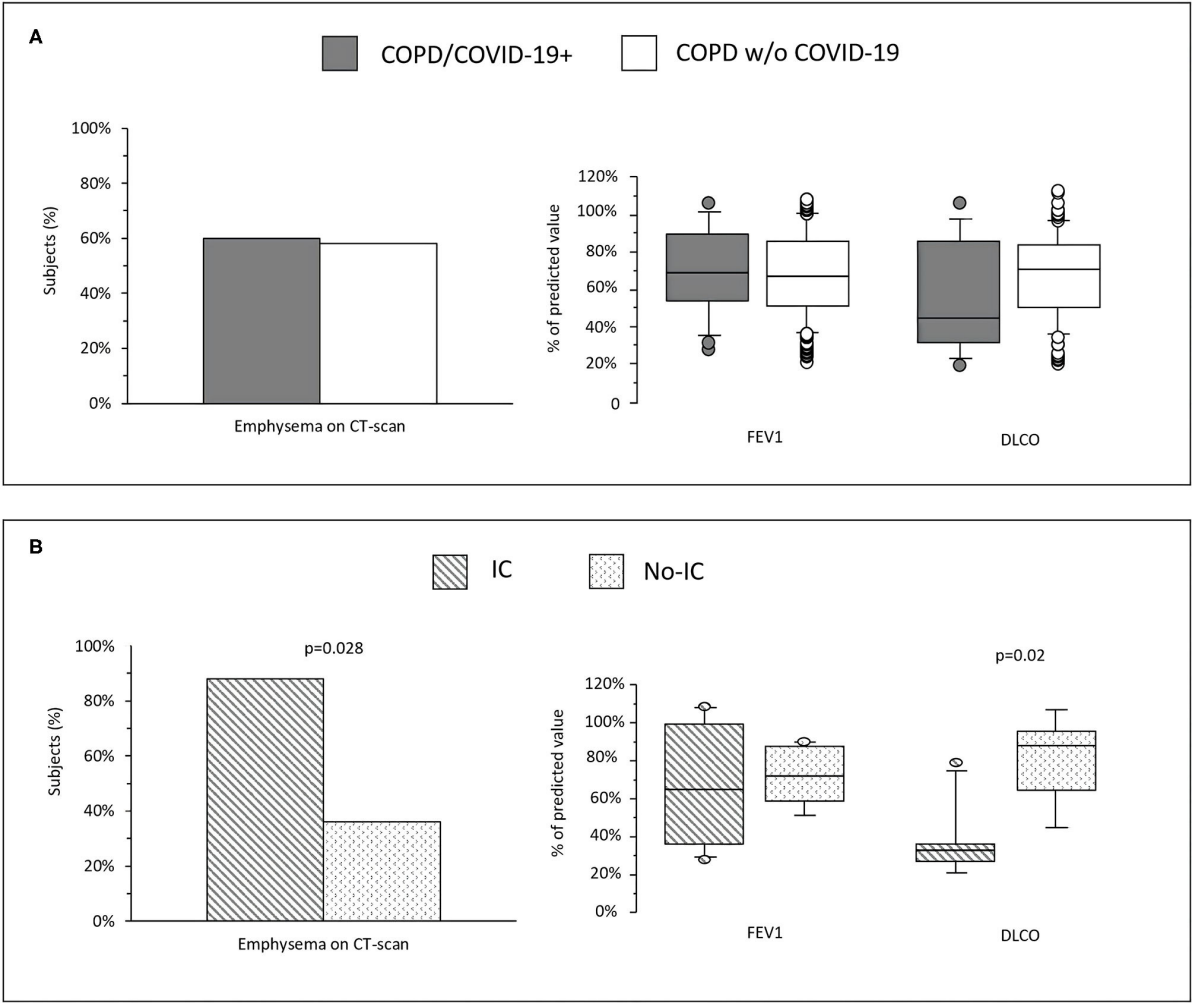
**(A)** Prevalence of emphysema and values of FEV_1_ and DLCO in COPD who developed COVID-19 (COPD/COVID-19+) and COPD without COVID-19 (COPD w/o COVID-19). **(B)** Prevalence of emphysema and values of FEV_1_ and DLCO in COPD/COVID-19+ subjects who needed intensive care (IC) and in those who did not (No-IC).

Twenty-two patients out of 370 (5.9%) developed COVID-19 (COPD/COVID-19+), while 348 did not (COPD w/o COVID-19). Table 1 shows that age, gender, and smoking history were similar in the two groups and that COPD/COVID-19+ had a higher prevalence of cardio-metabolic comorbidities (obesity, hypertension, diabetes, dyslipidemia, metabolic syndrome) compared to COPD w/o COVID-19. Conversely, lung function, presence of emphysema (Figure 1A), history of exacerbations, COPD treatment and blood counts were similar in the two groups (Table 1).

**Table 1:** Demographic, functional and clinical characteristics of COPD subjects who developed COVID-19 (COPD/COVID-19+) and COPD subjects who did not (COPD w/o COVID-19).

	**COPD/COVID-19+**	**COPD w/o COVID-19**	***p*-value**
Subjects, *n* (%)	22	348	–
Gender male, *n* (%)	16 (72)	247 (71)	n.s.
Age, years	79 (75–85)	77 (72–83)	n.s.
Smoking: current/ex/never, *n* (%)	4 (18)/18 (82)/0 (0)	94 (27)/243 (70)/11 (3)	n.s.
Smoking: pack-years	40 (19–51)	40 (22–51)	n.s.
Comorbidities, *n* (%)			
• Obesity	8 (36)	47 (14)	0.004
• Hypertension	22 (100)	207 (59)	<0.001
• Type 2 diabetes	9 (41)	62 (18)	0.002
• Dyslipidemia	14 (63)	76 (22)	<0.001
• Metabolic Syndrome	9 (41)	45 (13)	<0.001
FEV_1_, %pred.	69 (54–90)	67 (51–86)	n.s.
FEF_25−75_, %pred.	36 (27–54)	28 (18–44)	n.s.
DLCO, %pred.	45 (32–85)	71 (50–84)	n.s.
Blood eosinophils, cells/μL	120 (37–262)	180 (100–267)	n.s.
Blood lymphocytes, cells/μL	1,820 (1,445–2,302)	1,860 (1,430–2,427)	n.s.
AE in the previous year, *n*	0 (0–1)	0 (0–1)	n.s.
Emphysema on CT-scan^*^, *n* (%)	12 (60)	133 (58)	n.s.
	*n* = 20	*n* = 226	
Inhaler therapy, *n* (%)			
• None	4 (18)	16 (5)	n.s.
• LAMA or LAMA/LABA	9 (41)	164 (47)	n.s.
• LAMA/LABA/ICS or LABA/ICS or LAMA+LABA/ICS	9 (41)	168 (48)	n.s.

*Data refer to the last visit available before COVID-19*.

*Data are expressed as median (interquartile range) or absolute numbers (percent). The p-value is referred to the Mann-Whitney-U or Fisher Exact-tests comparisons, as appropriate*.

* *Data available on 246 out of 370 patients*.

*FEV_1_, Forced Expiratory Volume in the 1st s; FEF_25−75_, forced mid-expiratory flow; DLCO, diffusing capacity of the lung for carbon monoxide; AE, acute exacerbations; CT, computed tomography; LAMA, long acting muscarinic antagonist; LABA, long-acting beta2-agonist; ICS, inhaled corticosteroid*.

Out of the 22 COPD/COVID-19+ subjects, 20 (90%) were hospitalized and 2 (10%) home treated. Among hospitalized patients, 10 (50%) needed ICU. Table 2 shows the characteristics of subjects needing (IC) or not needing ICU (No-IC). Dyslipidemia and metabolic syndrome were significantly more prevalent in IC patients than in No-IC. FEV_1_ was not related to the need for ICU, however IC subjects had lower DLCO at baseline and higher prevalence of emphysema (Figure 1B). When first seen at Emergency Room, PaO_2_/FiO_2_ Ratio was the only parameter associated to IC and it was inversely related with hospitalization length (*r* = −0.63; *p* = 0.013) (Table 2). In hospital mortality rate was 18.1% (4/22), three patients died in IC and 1 in No-IC. Among in-hospital deceased patients, 3 of 4 had emphysema and a very low DLCO (median 35%pred.) even if with mild obstruction (median FEV_1_ 76%pred). At 6-months, follow-up mortality raised to 36.3% (8/22), 4 patients in IC group (40%), 4 in no-IC (33.3%).

**Table 2:** Demographic, functional and clinical characteristics of COPD subjects who developed COVID-19 categorized in subjects who needed intensive care (IC) and in those who did not (No-IC).

	**COPD/COVID-19+**		***p*-value**
	**IC**	**No-IC**	
Subjects, *n* (%)	10	12	
Gender male, *n* (%)	7 (70)	9 (75)	n.s.
**BASELINE**			
Age, years	78.2 (75.9–80)	79 (72–86)	n.s.
Smoking: current/ex, *n* (%)	2 (20)	2 (16)	n.s.
Smoking: pack-years	45 (32–57.5)	32 (15–45)	n.s.
Comorbidities, *n* (%)			
• Obesity	6 (60)	2 (16.6)	n.s. (0.074)
• Hypertension	10 (100)	12 (100)	n.s.
• Type 2 diabetes	6 (60)	3 (25)	n.s. (0.1)
• Dyslipidemia	9 (90)	5 (41.6)	0.032
• Metabolic Syndrome	7 (70)	2 (16.6)	0.027
FEV_1_, %pred.	65 (36.5–99.5)	72 (58.7–87.7)	n.s.
FEF_25−75_, %pred.	39 (28–77)	36 (26–52.7)	n.s.
DLCO, %pred.	32 (28–35)	88 (64.5–95.7)	0.02
Blood eosinophils, cells/μL	110 (10–270)	120 (90–205)	n.s.
Blood lymphocytes, cells/μL	1,755 (1,450–2,290)	1,820 (1,440–2,325)	n.s.
AE in the previous year, *n*	0.5 (0–2)	0 (0–0.5)	n.s.
Emphysema on CT-scan, *n* (%)^*^	8 (88.8)	4 (36.3)	0.028
	*n* = 9	*n =* 11	
Inhaled therapy, *n* (%)			
• None	1 (10)	3 (25)	n.s.
• LAMA or LAMA/LABA	5 (50)	4 (33)	n.s.
• LAMA/LABA/ICS or LABA/ICS or LAMA+LABA/ICS	4 (40)	5 (42)	n.s.
**ON ADMISSION**			
P/F ratio	180 (124–296)	310 (262–364)	0.02
C-Reactive protein, mg/dl	39 (18–117)	43 (17–130)	n.s.
D-dimer, ng/L	238 (167–669)	258 (181–596)	n.s.
BNP, ng/L	224 (94–319)	174 (100–201)	n.s.
Blood lymphocytes, cells/μL	815 (570–1,590)	695 (610–890)	n.s.
**OUTCOME**			
Hospitalization length, days (all patients)	21 (8–46)	8.5 (6–11.12)	0.05
Hospitalization length, days (discharged alive only)	36 (13.7–53.5)	8 (5.25–10.5)	0.01
In-hospital mortality, *n* (%)	3 (30)	1 (8)	n.s.
6-month mortality, *n* (%)	4 (40)	4 (33)	n.s.

*Baseline data refer to the last visit available before COVID-19*.

*Data are expressed as median (interquartile range) or absolute numbers (percent). The p-value is referred to the Mann-Whitney-U or Fisher Exact tests comparisons as appropriate*.

* *Data available on 20 out of 22 patients. IC: subjects who needed intensive care. No-IC: subjects who did not need intensive care*.

*FEV_1_, Forced Expiratory Volume in the 1st s; FEF_25−75_, forced mid-expiratory flow; DLCO, diffusing capacity of the lung for carbon monoxide; AE, acute exacerbations; CT, computed tomography; LAMA, long acting muscarinic antagonist; LABA, long-acting beta2-agonist; ICS, inhaled corticosteroid; P/F, PaO_2_/FiO_2_ ratio; BNP, brain natriuretic peptide*.

## Discussion

Our study shows that the incidence COVID-19 in a cohort of longitudinally followed COPD subjects, was 5.9% [the incidence in Italian population older than 60 years in the same period was 2.8% (5)]. To our knowledge, this is the first time the incidence of COVID-19 in a cohort of COPD patients is described. Only the incidence of COPD in COVID-19 cohorts had been previously reported (2). Despite the small sample size, longitudinal data before COVID-19 allowed the evaluation of the possible risk and prognostic factors in our cohort.

Among the possible risk factors for the development of COVID-19, we found that patients who developed COVID-19 had higher prevalence of hypertension, obesity, type-2 diabetes and dyslipidemia than those who did not. It is noteworthy that, even in a cohort where comorbidities are highly frequent, their prevalence was still significantly higher in subjects who developed COVID-19. This was particularly relevant for hypertension, that was present in all COVID-19+ subjects, reinforcing the known association between this comorbidity and COVID-19 (6). Of interest, we found that functional and respiratory risk factors (FEV_1_, DLCO, exacerbations) were not associated to development of COVID-19 in COPD patients.

When the prognostic factors predicting the severity of COVID-19, defined as the need for intensive care, were assessed, we found that the presence of metabolic comorbidities was associated with more severe COVID-19, as in the general population (7). In addition, we observed that the presence of emphysema and low DLCO were associated with the need for ICU, while FEV_1_ was not. Our observations suggest that, in a context of mild-moderate airflow obstruction, the severity of COVID-19 could be dependent on a decreased DLCO, which reflects a previous impairment of the alveolar-capillary unit secondary to impaired perfusion due to microvascular destruction in emphysema and to compression of the intra-alveolar pulmonary vessels by gas trapping, rather than on the degree of airflow obstruction. The vascular abnormalities reflected by the DLCO in COPD would compound the severity of COVID-19, a disease with an important pulmonary vascular disorder with diffused micro thrombosis or intrapulmonary shunting inducing what has been defined an acute vascular distress syndrome (AVDS) (8). Indeed, there is increasing evidence that the virus can assault directly the capillary endothelium causing diffused micro thrombosis, destroying the lung vascular bed and increasing the dead space (9). Of interest, in a recent follow-up of COVID-19 subjects, patients hospitalized with severe disease had lower DLCO at 3–4 months post-infection compared to those non-hospitalized, probably reflecting the SARS-CoV-2 induced pulmonary vascular disorder, while the FEV_1_ was similar (10). Unfortunately, no baseline DLCO was available. If COVID-19 worsens DLCO, it would be expected that a low baseline DLCO ought to be an important risk for severe respiratory failure. Our data points to the value of a baseline DLCO in patients with COPD as a useful predictor of the disease outcome.

Our study has some limitations: first the small sample size did preclude more extensive analyses. Furthermore, our study included only patients presenting at the hospitals for symptomatic COVID-19, which relates to the high hospitalization rate observed in these patients. Although we cannot exclude that mild symptomatic patients were tested outside the public health system, it seems unlikely that patients with chronic respiratory diseases would seek medical care outside of their referral center. Finally, the characterization of our cohort is based on data collected during the routine assessment of COPD patients at outpatient clinics, therefore we did not have any standardized emphysema quantification on CT scan.

In conclusion, in our cohort of COPD subjects, 5.9% developed COVID-19. Cardiometabolic, but not respiratory abnormalities, were risk factors for the development of COVID-19. A low DLCO and the presence of emphysema, but not FEV_1_, were prognostic factors for worse outcomes of COVID-19 in COPD. These results highlight the importance of preventive measures and social distancing in patients with COPD with cardiometabolic comorbidities, at risk of developing COVID-19. Moreover, in COPD patients with COVID-19, a low DLCO and emphysema herald unfavorable outcomes, which advises close monitoring in these subjects.

## Data Availability Statement

The raw data supporting the conclusions of this article will be made available by the authors, without undue reservation.

## Ethics Statement

Ethical review and approval was not required for the study on human participants in accordance with the local legislation and institutional requirements. The patients/participants provided their written informed consent to participate in this study.

## Author Contributions

MB, US, MR, GT, MC, MS, and SB: contributed to conception and design of the study and drafting, and editing the manuscript. MT, EB, MD, DB, and AC: undertook data collection and performed data analysis. MB, US, EB, MR, GT, MC, MS, and SB: data management and data interpretations. All authors critically revised the manuscript for important intellectual content and approved the final version.

## Conflict of Interest

The authors declare that the research was conducted in the absence of any commercial or financial relationships that could be construed as a potential conflict of interest.

## Publisher's Note

All claims expressed in this article are solely those of the authors and do not necessarily represent those of their affiliated organizations, or those of the publisher, the editors and the reviewers. Any product that may be evaluated in this article, or claim that may be made by its manufacturer, is not guaranteed or endorsed by the publisher.
